# WITHDRAWN: A Machine Learning Model for Real-Time Hypoglycemia Risk Prediction in Hospitalized Diabetic Patients: Development and Validation

**DOI:** 10.21203/rs.3.rs-6171081/v2

**Published:** 2025-04-16

**Authors:** Liren Li, Jiayu Chen, Tongshu Guan, Ze Yu, Jinyuan Zhang, Ronghong Ji, Zike Li, Ming Lei, Ping Zheng, Yilei Li, Fei Gao

## Abstract

The full text of this preprint has been withdrawn, as it was submitted in error. Therefore, the authors do not wish this work to be cited as a reference. Questions should be directed to the corresponding author.

